# The Role of SilX in Bacteriocin Production of *Streptococcus anginosus*

**DOI:** 10.3389/fmicb.2022.904318

**Published:** 2022-07-01

**Authors:** Verena Vogel, Miki Fuchs, Marie Jachmann, Alina Bitzer, Stefanie Mauerer, Jan Münch, Barbara Spellerberg

**Affiliations:** ^1^Institute of Medical Microbiology and Hygiene, Ulm University Medical Center, Ulm, Germany; ^2^Institute of Molecular Virology, Ulm University Medical Center, Ulm, Germany

**Keywords:** *Streptococcus anginosus*, CAAX protease, bacteriocin, Angicin, streptococcal invasion locus, HIV protease inhibitor

## Abstract

*Streptococcus anginosus* produces the novel antimicrobial peptide Angicin, which inhibits Gram positive microorganisms and is classified as a group IId bacteriocin. Production of Angicin is regulated by the quorum sensing system *Sil* (Streptococcus invasion locus), which is located adjacent to the bacteriocin gene cluster. Within this genetic region a typical CAAX protease is encoded, which was designated SilX. Nelfinavir, a HIV protease inhibitor, led to a concentration dependent reduction in antimicrobial activity, presumably through the inhibition of SilX. Concentrations exceeding 25 μM Nelfinavir caused a complete abolishment of bacteriocin activity against *Listeria monocytogenes*. These results are supported by the observation, that a SilX deletion mutant of *S. anginosus* strain BSU 1211 no longer inhibits the growth of *L. monocytogenes.* Antimicrobial activity could be restored by addition of synthetically synthesized mature SilCR, implying that SilX may be involved in the export and processing of the signal peptide SilCR. Some CAAX proteases have been reported to provide immunity against bacteriocins. However, in a radial diffusion assay the deletion mutant *S. anginosus* BSU 1211ΔSilX showed no sensitivity toward Angicin arguing against a role of SilX in the immunity of *S. anginosus*. The putative processing of the signal peptide SilCR indicates a novel function of the CAAX protease SilX, in the context of *S. anginosus* bacteriocin production.

## Introduction

Bacterial communication through quorum sensing (QS) represents a major regulatory mechanism for group behavior and bacteriocin production. Bacteriocins are small antimicrobial peptides of bacterial origin inhibiting the growth of other often closely related bacterial species. Producing bacteriocins provides a colonization advantage and influences the composition of microbial communities (Majeed et al., 2011; Thappeta et al., 2020; Vogel and Spellerberg, 2021). It is therefore critical to understand how bacteriocin production is regulated and which genes are required for bacteriocin production. CAAX proteases are often present in bacteriocin clusters. While they have repeatedly been implicated in immunity, their precise function in bacteriocin production is not clearly understood, for many species.

CAAX proteases are multipass, transmembrane proteins that contain two typical sequence motifs (EExxxR, FxxxH) that are necessary for the catalytic activity (Pei et al., 2011). The function of type II CAAX proteases in bacteria is ambiguous. CAAX proteases have been demonstrated to confer immunity to bacteriocins, process bacteriocins or function as a receptor for bacteriocins (Diep et al., 1996; Lux et al., 2007; Kjos et al., 2010; Biswas and Biswas, 2014; Maxson et al., 2015). For example, in the hemolysin gene cluster of *Streptococcus pyogenes*, SagE supposedly cleaves off the leader peptide of SagA, leading to the formation of the hemolysin StreptolysinS, the mature form of SagA (Maxson et al., 2015). SagE has previously been annotated as a bacteriocin self-immunity protein against Streptolysin, however, no clear evidence supporting this hypothesis has been found (Maxson et al., 2015). As another example, LsrS seems to have a receptor-like function for the two-peptide bacteriocin Smb produced by *Streptococcus mutans* (Biswas and Biswas, 2014). Overexpression of LsrS rendered cells more sensitive toward Smb, while inactivation led to Smb resistance. However, not the active CAAX domain is responsible for this phenotype since inactivation of this site had no effect on Smb sensitivity.

Besides their roles in bacteriocin production, CAAX proteases can interfere with QS systems. An interaction of CAAX proteases with histidine kinases has already been demonstrated. In these cases, the catalytically necessary motifs are not essential, e.g., SpdC, a CAAX protease of *Staphylococcus aureus*, interacts with various histidine kinases of this pathogen but lacks all conserved catalytic domains (Poupel et al., 2018). Furthermore, for *Streptococcus agalactiae* it was demonstrated that the CAAX protease Abx1 forms a signaling complex with histidine kinase CovS (Firon et al., 2013). There is a direct interaction between the transmembrane domain of CovS and Abx1, which is independent from its proteolytic activity. CovS forms together with CovR a two-component system, which regulates virulence and hemolysis in *S. agalactiae* (Lamy et al., 2004; Rosa-Fraile et al., 2014). The conserved catalytic motifs are present in Abx1 but are not necessary for its effect on CovS. In contrast to this, the conserved catalytic motifs are important for the activity of MroQ, a CAAX protease of *Staphylococcus aureus*. MroQ facilitates the maturation of the autoinducing peptide (AIP) of the accessory gene regulatory system (Agr) in *S. aureus* and thereby controls the expression of virulence factors (Le and Otto, 2015; Cosgriff et al., 2019).

A putative CAAX protease has also been identified in *Streptococcus anginosus* in the context of bacteriocin production (Vogel et al., 2021). *S. anginosus* is primarily a commensal of mucosal membranes. For a long time, the pathogenic potential of this species was underestimated, due to diagnostic difficulties in distinguishing the species from other viridans streptococci (Facklam, 2002; Asam and Spellerberg, 2014). As an opportunistic pathogen this species can cause severe invasive and pyogenic infection. Frequent isolation from abscesses, blood cultures and cystic fibrosis patients has been reported in more recent years (Siegman-Igra et al., 2012; Kobo et al., 2017; Jiang et al., 2020). We previously identified a novel bacteriocin of *S. anginosus* termed Angicin (Vogel et al., 2021). Angicin is a class IId bacteriocin and active against other streptococci as well as against *Listeria spp.* and enterococci. The QS system *Sil* (Streptococcal invasion locus) regulates bacteriocin production in *S. anginosus* and *Streptococcus intermedius* (Mendonca et al., 2016; Vogel et al., 2021). SilCR, the autoinducing peptide, is processed and exported by SilD and SilE (Belotserkovsky et al., 2009). Extracellular SilCR is then sensed by the histidine kinase SilB, which in turn phosphorylates the response regulator SilA. SilA induces gene expression of the *sil* genes as well as of the adjacent bacteriocin genes. A putative CAAX protease gene, designated silX, is located in the vicinity of the bacteriocin genes (*blp3*) and the *sil* genes of *S. anginosus* (Vogel et al., 2021). Whether this protease plays a part in self immunity, processing or as a bacteriocin receptor is unknown. To better understand the biosynthesis and regulation of Angicin production the role of SilX was investigated.

## Materials and Methods

### Bacterial Strains and Growth Conditions

Lysogeney Broth (LB-Miller) was used to cultivate *Escherichia coli* DH5α. Liquid *E. coli* cultures were incubated aerobically at 37°C on a shaker (180 rpm), whereas plates were kept at 37°C with 5% CO_2_. Two plasmid backbones, pAT28 and pAT18 were used in this study. 100 μg ml^–1^ spectinomycin (pAT28) (Sigma-Aldrich Chemie GmbH) or 400 μg mg^–1^ erythromycin (pAT18) (Serva) were supplemented for cultivation of *E. coli* strains carrying these plasmids. For cultivation on solid media, all bacterial stains were cultivated on sheep blood agar plates (Oxoid). For streptococci and listeria strains a liquid culture in THY medium [Todd-Hewitt Broth (Oxoid) supplemented with 0.5% yeast extract (BD)] was prepared. Liquid cultures as well as plates were incubated at 37°C and 5% CO_2_. Streptococcal mutants were incubated with 120 μg ml^–1^ spectinomycin or 10 μg ml^–1^ erythromycin if necessary. All strains and plasmids used in this study are summarized in Table 1.

**TABLE 1:** Strains and plasmids used in this study.

Strain or plasmid	Definition	Source
*Escherischia coli* DH5α	*endA1 hsdR17 supE44* DlacU169(f80lacZDM15) *recA1 gyrA96 thi-1 relA1*	Boehringer
*Streptococcus anginosus* SK 52	*S. anginosus* type strain, ATCC 33397, Hly +	ATCC
*Streptococcus anginosus* BSU 1211*^a^*	*S. anginosus*, clinical isolate	Bauer et al., 2020
*Streptococcus anginosus* BSU 1401*^a^*	*S. anginosus*, clinical isolate	Bauer et al., 2020
*Streptococcus constellatus* BSU 1213*^a^*	*S. constellatus*, clinical isolate	Vogel et al., 2021
*Listeria monocytogenes* EGDe*^b^*	Ln II Serotype I/2a	Bécavin et al., 2014
**Mutants**		
*Streptococcus anginosus* BSU 1211ΔCAAX	This study	
*Streptococcus anginosus* SK 52 + pBSU 100		Aymanns et al., 2011
*Streptococcus anginosus* SK 52 + *pAT28_silXprom_EGFP*		This study
*Streptococcus anginosus* SK 52 + pBSU 101		Aymanns et al., 2011
**Plasmids**		
pAT28	lacZα, ori pUC, ori pAmβ1, Spc*^R^*	Trieu-Cuot et al., 1990
pBSU 100	pAT28 derivate carrying *egfp*	Aymanns et al., 2011
pBSU 101	pAT28 derivate carrying *egfp* under the control of the *cfb* promoter	Aymanns et al., 2011
pBSU 1202	pAT28 derivate carrying *egfp* under the control of the *silX* promoter	This study
pAT18	pAT18-lacZα, ori pUC, ori pAmβ1, Em*^R^*	Trieu-Cuot et al., 1991
pAT18-cre-rec_*tufA*_	pAT18 derivative carrying Cre-recombinase gene under the control of *tufA* promoter, Em*^R^*	Bauer et al., 2018

*^a^Isolated at Ulm University Medical center, Ulm, Germany. ^b^Kindly provided by Prof. Dr. C. Riedel, Ulm University, Ulm, Germany.*

### General DNA Techniques

To isolate DNA (GeneEluteTM Bacterial Genomic DNA, Sigma-Aldrich; QIAamp^®^ DNA Mini Kit, Qiagen) or plasmids (QIAprep^®^ Spin Miniprep Kit, Qiagen) commercial kits were applied, following the manufacturer’s protocols. QIAamp DNA Mini Kit was used to screen clones for successful transformation, while GeneEluteTM Bacterial Genomic DNA was the standard DNA isolation kit. DNA and plasmid concentrations were measured using Quant-iT dsDNA Broad-Range (BR) Assay Kit (Invitrogen). Polymerase chain reactions (PCR) were conducted following standard protocols for *Taq* polymerase (Roche) or the Expand Long Template PCR system: DNA pol. Mix (Roche) with buffer 3. For *Taq* polymerase an initial denaturation at 95°C for 5 min was followed by 32 cycles of 1 min at 95°C, 30 s at 50°C and 1–3 min at 72°C. A final elongation of 7 min at 72°C was the last step. Elongation time was adjusted according to product length (1 min per 1ooo basepairs) Default settings for the Long Expand PCR were an initial denaturation for 5 min at 92°C followed by 11 cycles of 92°C for 10 s, 50°C for 20 s and 68°C for 3 min. This was followed by 25 cycles of 92°C for 15 s, 50°C for 20 s and 68°C for 3 min + 25 s per cycle. It was rounded up by a final elongation of 68°C for 5 min. Clean up of PCR products was performed using NucleoSpin Gel and PCR Clean-up (Machery-Nagel). All Primers used for this study are summarized in Table 2. Nucleotide sequencing was performed by Eurofins Genomics (Germany) and Microsynths laboratories (Switzerland).

**TABLE 2:** Primers used in this study.

Primer name	Sequence (5′-3′)	Nr.
SilX_F1_fwd	agtatgttaatcgctctaatc	1
SilX_F1_rev	gcatacattatacgaacggtaccaaatgatagcgttggtgtc	2
SilX_F2_fwd	tataatgtatgctatacgaacggtcatagcctctatctggtatatc	3
SilX_F2_rev	caattcagagtgactggttac	4
lox71_spec_fwd	taccgttcgtatagcatacattatacgaagttatttaaatggcattggtaccc	5
lox66_spec_rev	taccgttcgtataatgtatgctatacgaagttatatgcctgcaggtcgattttcg	6
Spec_fwd	gtaaccattctccataaataaattc	7
SilX_del_fwd	gaggtctattctgtttgtatg	8
SilX_del_rev	cagtatgtagcagcgcagtttc	9
SilCR_F1_fwd	gtccttatcgcttattatgtg	10
SilCR_F1_rev	gcatacattatacgaacggtacaccgacaacttgttcgagttc	11
SilCR_F2_fwd	tataatgtatgctatacgaacggtagtacttgatattacagcaatgg	12
SilCR_F2_rev	gttttcgcaatcccaagtatg	13
SilCR_del_fwd	ctttcggcatgattacgcta	14
SilCR_del_rev	gattagtcgctcccgaaacag	15
SilX_*Eco*RI_promEGFP_fwd	ggcgcgggatccagtgaaaatgctaatttct	16
SilX_*Bam*HI_promEGFP_rev	gccgcggaattcgtacttctgacatcctgaatc	17
pAT28-3	gttgtgtggaattgtgagcgg	18
pAT28-2	ctcttcgctattacgccagct	19
pAT28EGFP-4	ccttgaagaagatggtgcgc	20

### Construction of Mutants

#### Construction of CAAX Deletion Mutants

For creating markerless deletion mutants the natural competence system of *S. anginosus* was exploited as described by Bauer et al. (2018). In a first step a splicing by overlap extension PCR (SOE-PCR) was performed. Flanking regions of the target gene, *CAAX*, were amplified with primers 1/2 for fragment 1 and 3/4 for fragment 2. Primers 2/3 introduced an overlap to either a *lox66* or a *lox71* sequence. Primers 5/6 were used to amplify a spectinomycin resistance gene from pGA14-spec and to simultaneously introduce a *lox66* and a *lox71* site adjacent to the spectinomycin gene. All three fragments were fused together by SOE-PCR. The fusion construct was loaded on an agarose gel (1%) to control for the right size. Subsequently, this linear construct was transformed into *S. anginosus. S. anginosus* BSU 1211 was incubated with 100 ng of competence-stimulating peptide 1 (CSP-1) to induce natural competence. After 40 min of incubation at 37°C the linear construct was added, and it was incubated for another 80 min at 37°C and subsequently plated on THY-spectinomycin agar. Colonies were picked after 24–48 h and screened for successful insertion with a colony-PCR. Therefore, streptococci were resuspended in 50 μl Aqua bidest and then incubated at 95°C for 30 min. Subsequently, the supernatant was used in a standard *Taq*-PCR (see “General DNA Techniques”). Primers 1/7 were used to amplify the DNA present in the supernatant. Positive clones were transformed with a Cre-recombinase harboring plasmid (pAT18-cre-rectufA) using the above-described method. In this case, transformed cells were plated on sheep blood agar plates supplemented with 10 μg ml^–1^ erythromycin The Cre-recombinase recombines the two lox sites to a singular *lox72* site and thereby eliminates the spectinomycin resistance gene. Spectinomycin-sensitive but erythromycin resistant clones were incubated without antibiotics to induce plasmid loss resulting in a markerless deletion strain. The created deletion was amplified by PCR using primers 8/9 and checked by subsequent DNA sequencing.

The same method was used to create a markerless SilCR knockout strain of *S. anginosus* BSU 1211. Primers 10/11 (F1) and 12/13 (F2) were used to create the flanking fragments and primers 14/15 were used to verify a correct deletion.

#### EGFP Expressing Mutants

To analyze SilX expression a reporter construct with EGFP was created. The putative SilX promotor was amplified using primers 16/17, which introduced restriction cutting sites for *Bam*HI and *Eco*RI. Both enzymes (New England Biolabs) were used to digest the PCR product as well as plasmid pBSU101. After purification both products were ligated and transformed into *E. coli* DH5α by heat shock transformation. Plasmid DNA was extracted and used for a transformation into *S. anginosus* strain BSU 1211 via inducing natural competence as described by Bauer et al. (2018). Correct plasmid construction was controlled by PCR using primers 18/19/20 and subsequent DNA sequencing.

### Radial Diffusion Assay

Bacteriocin activity was quantified using a modified radial diffusion assay (RDA) (Vogel et al., 2021). Briefly, overnight cultures of putative target strains were washed and O.D._600 *nm*_ was determined. Per plate 2 × 10^7^ bacterial cells were seeded. After washing with 10 mM Phosphatebuffer, overnight cultures of bacteriocin producing *S. anginosus* strains were adjusted to an O.D._600 *nm*_ of 0.5. With wide bore pipette tips (Ayxgen- A Corning Brand, Corning Inc., Salt Lake City, UT, United States) wells were placed into the agar plates of target strains, which were subsequently filled with the overnight cultures of bacteriocin producing strains. Following overnight incubation at 37°C and 5% CO_2_, inhibition zones were measured in cm.

In some assays 100 ng of the signal peptide SilCR_*SAG–C*_ (GWLEDLFSPYFKKYKLGKLGQPDLG) were added simultaneously with the bacteria to induce bacteriocin production. The peptide was synthesized by the Core facility of functional peptidomics (UPEP, Ulm University, Ulm, Germany) based on the deduced sequence of SilCR_*SAG–C*_ of *S. anginosus* strain BSU 1211.

In some experiments, the HIV protease inhibitors Saquinavir (Sigma-Aldrich) or Nelfinavir (Sigma-Aldrich) were administered in combination with bacteriocin producing bacteria. Dimethyl sulfoxid (DMSO, Sigma), in which both inhibitors were solved, was used as a control. Experiments were conducted in technical duplicates.

### FACS Analysis

To assess the activity of the *silX* gene the *silX* promoter of *S. anginosus* strain BSU 1211 was cloned in front of *egfp* to construct plasmid pBSU 1202. The newly constructed plasmid was transformed into strain *S. anginosus* SK52. After overnight incubation it was freshly inoculated in 3 ml of THY supplemented with 120 μg ml^–1^ spectinomycin either in the presence or absence of 10 μg ml^–1^ SilCR. After 2 h and cells were harvested by centrifugation (3000 × g, 10 min), washed, and resuspended in Dulbecco’s phosphate buffered saline (DPBS, pH 7.4). The mean fluorescence of 10 000 events was determined by flow cytometer. The FACSCalibur (Becton Dickinson Immunocytometry Systems) was utilized with the following instrument settings: FSC: E00, SSC: 400, FL-1: 700. As a control *egfp* without promoter and *egfp* under the control of the *cfb* promoter was used. All experiments were performed with technical triplicates.

### Bioinformatic and Statistical Analysis

The GenBank database^1^ was used as a source for nucleotide sequences. Homology searches were done with the Basic Local Alignment Search Tool (Altschul et al., 1990)^2^. Other genetic analysis was carried out using SnapGene 5.0^3^. Phyre2 was used to analyze the SilX protein sequence (Kelley et al., 2015). An alignment of putative *silA* binding sites was constructed using CLC Main Workbench v7.7.3^4^ with default settings (gap open cost value 10.0, gap extension cost value 1.0). Preparation of graphs and statistical analysis was performed by GraphPad Prism V6 (GraphPad Software Inc., La Jolla, CA, United States).

## Results

### In Silico Analysis of SilX in *Streptococcus anginosus*

**FIGURE 1:**
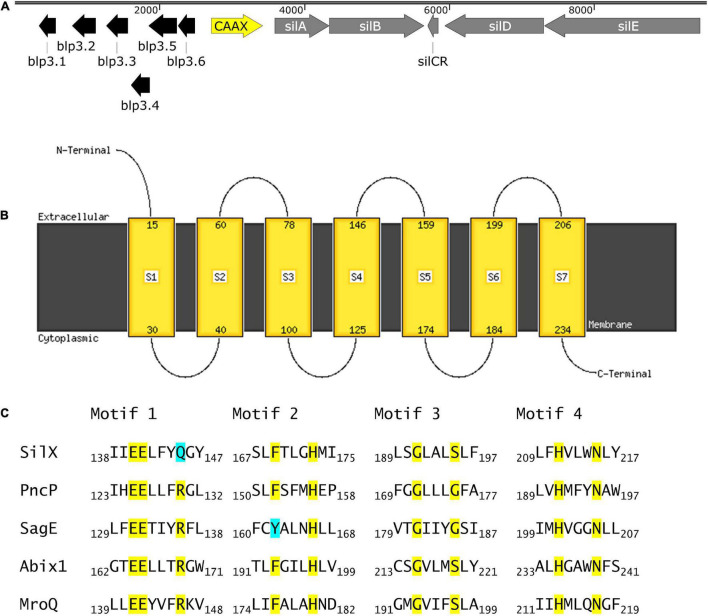
SilX is a transmembrane protein harboring conserved CAAX domains. **(A)** Genetic organization of the bacteriocin and quorum sensing locus in *S. anginosus* BSU 1211 (Accession: MZ766502). Modified from Vogel et al. (2021). **(B)** Prediction of protein transmembrane structure of SilX constructed using Phyre2. **(C)** SilX is a member of the CAAX proteins. Conserved domains of CAAX proteins are depicted with the important residues marked in yellow and differing residues marked in turquoise. SilX is compared to PncP of *Streptococcus mitis* (Acsession number: VTS34806.1), SagE of *S. pyogenes* (Accession number: QJC39319.1), Abix1 of *Streptococcus agalactiae* (Accession number: WP_001042316.1) and MroQ of *S. aureus* (Accession number: ABD20720.1).

*SilX* is located between the response regulator *silA* of the QS system Sil and the bacteriocin-like-peptide3 (*blp3*) region (Figure 1A), which encodes for the functional bacteriocin Angicin of *S. anginosus* (Vogel et al., 2021). The *silX* gene has the same orientation as *silA* and the deduced amino acid sequence displays homologies to immunity proteins and streptococcal metalloproteases, while its function has not been investigated. By Blast analysis *silX* homologs can only be found in the *Streptococcus anginosus* Group, while *Streptococcus constellatus* shows a higher percentage of sequence identity (98.74%) than *Streptococcus intermedius* (97.35%).

To further characterize SilX its protein structure was predicted using Phyre2. This analysis revealed CAAX protease motifs with close homologies to other streptococcal and staphylococcal CAAX proteases. Seven transmembrane domains could be detected with the C-terminus of SilX being on the cytoplasmic site and the N-termiuns reaching into the extracellular lumen (Figure 1B). Most of the known motifs of CAAX proteases are conserved in SilX, however, in the diglutamate motif (EExxxR) the arginine residue is replaced by a glutamine (EExxxQ) (Figure 1C). The diglutamate motif (E140-E141) has been reported as necessary for the catalytic function of CAAX proteases (Pei et al., 2011). In summary, the *in silico* analysis supports a putative function of SilX as a CAAX protease.

### The Role of SilX in *Streptococcus anginosus* Bacteriocin Production

**FIGURE 2:**
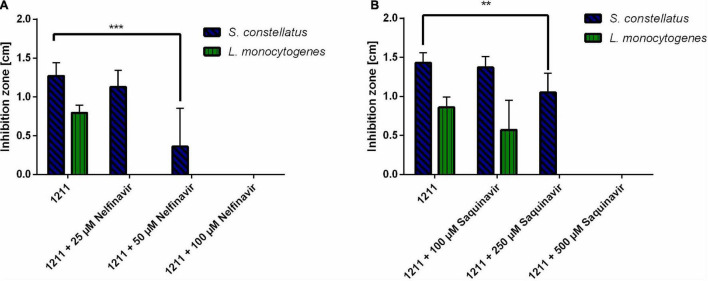
HIV protease inhibitors inhibit Angicin activity of *S. anginosus*. The antimicrobial activity of *S. anginosus* BSU 1211 against *S. constellatus* and *L. monocytogenes* was tested in an RDA. *S. anginosus* BSU 1211 was supplemented with **(A)** Nelfinavir concentrations ranging from 25 to 250 μM or **(B)** Saquinavir concentrations ranging from 100 to 500 μM. Depicted is inhibition zone diameter ± standard deviation of at least five independent experiments. Significance was calculated using Mann-Whitney U test with * presenting a *p*-value < 0.5, ** indicating *p* < 0.01 and *** illustrating p < 0.001.

It has previously been shown that HIV protease inhibitors are able to inhibit CAAX proteases, like SagE of *Streptococcus pyogenes* (Maxson et al., 2015). To explore, if a similar effect can be achieved for SilX we investigated the effect of HIV protease inhibitors Nelfinavir and Saquinavir on the bacteriocin production of *S. anginosus.* Bacteriocin activity of *S. anginosus* BSU 1211 against two highly susceptible target strains (*Streptococcus constellatus, Listeria monocytogenes*) was tested in a radial diffusion assay (RDA) either in the presence or absence of HIV protease inhibitors (Figure 2).

The supplementation with Nelfinavir or Saquinavir caused a dose dependent reduction of bacteriocin activity. At concentrations of 250 μM Saquinavir *S. anginosus* BSU 1211 was no longer able to inhibit the growth of *L. monocytogenes*, while 500 μM Saquinavir were needed to completely abolish the antimicrobial effect against *S. constellatus* (Figure 2A). In the case of Nelfinavir, already 25 μM were sufficient to abolish antimicrobial activity against *L. monocytogenes* (Figure 2B). For a complete loss of inhibition against *S. constellatus* 100 μM of Nelfinavir are needed.

**FIGURE 3:**
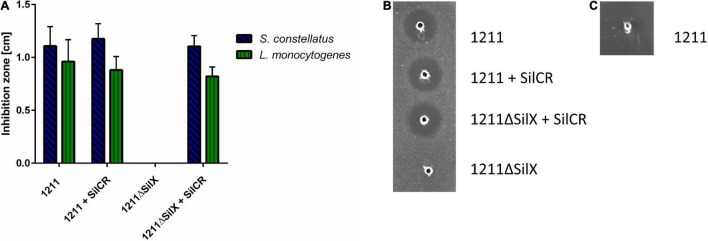
*S. anginosus* BSU 1211ΔSilX has no antimicrobial activity against *S. constellatus* and *L. monocytogenes*. A SilX deletion mutant of *S. anginosus* was tested in a RDA against *S. constellatus* and *L. monocytogenes* either alone or supplemented with 10 μg x ml^– 1^ SilCR. **(A)** Depicted is the mean of inhibition zone diameter ± standard deviation of at least 5 independent experiments. No significant differences were found with a Mann-Whitney-U test. **(B)** Depicted is a RDA of *S. anginosus* BSU 1211 and *S. anginosus*ΔSilX with *S. constellatus* as target strain under supplementation with 10 μg x ml^– 1^ SilCR as indicated. **(C)** Depicted is a RDA of *S. anginosus* BSU1211ΔSilX as a target strain against *S. anginosus* BSU1211.

To investigate, if the gene *silX* is required for effective bacteriocin production of *S. anginosus*, *silX* of strain BSU1211 was deleted and the mutant strain was tested for antimicrobial activity in a RDA (Figure 3). *S. anginosus* BSU 1211ΔSilX demonstrated a complete loss of growth inhibition for the species *L. monocytogenes* and *S. constellatus.* This observation indicates an essential role of SilX in bacteriocin production. However, the antimicrobial activity of the deletion mutant could be reestablished to previous levels by supplementation with the signaling peptide SilCR at a concentration of 10 μg × ml^–1^.

To investigated whether SilX may also serve as a immunity protein *S. anginosus* BSU 1211ΔSilX was checked for bacteriocin susceptibility toward the wildtype strain BSU 1211. The deletion mutant showed no sensitivity toward the wildtype strain arguing against an essential role in bacteriocin immunity. In conclusion our data support an essential role of the presumed protease function of SilX for bacteriocin activity, which can however be substituted by supplementation with the signal peptide SilCR.

### SilX Promoter

**FIGURE 4:**
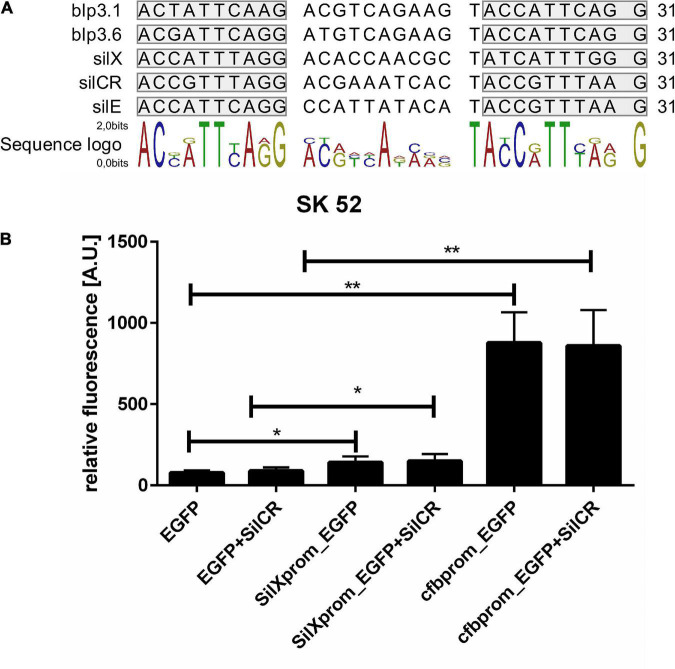
Identification and activity of the SilX promotor. **(A)** Predicted SilA binding sites. Overview was constructed using CLC main workbench 7. Imperfect repeats are marked in gray. **(B)** Depicted is the mean fluorescence of *S. anginosus* SK52 **(B)** transformed with *egfp* without promoter (negative control), with the SilX promoter or the *cfb* promoter (positive control) either with or without 10 mg/l synthetic SilCR. Depicted are means + standard deviation of five independent experiments. Significant differences were calculated using a Man-Whitney U test with * illustrating a *p*-value < 0.5 and ** indicating *p* < 0.01.

The gene *silX* in *S. anginosus* is located adjacent to the *streptococcal invasion locus* (*sil*) (Figure 1A) and transcribed in the same orientation as the genes *silA* and *silB*. *SilA* encodes a response regulator belonging to the AlgR/AgrA/LytR family of transcription regulators that bind DNA via a LytTR-type domain (Nikolskaya, 2002; Belotserkovsky et al., 2009). Since these regulators form dimers and typically bind to direct repeats, we searched for this motif upstream of *silX*. A putative promoter could be located in this region, consisting of 11 bp long imperfect repeats interspaced with 10 bp. Similar putative promoters were identified in front of *silCR* and *silE* as well as upstream of the putative bacteriocin genes *blp3.1* and *blp3.6* (Figure 4A).

To assess the activity and functionality of the putative SilX promoter, it was cloned in front of an EGFP gene on the plasmid reporter gene construct pBSU 1202 and transformed into *S. anginosus*. As a control, the *egfp* gene alone (pBSU 100) or under the control of an overexpression promoter (pBSU 101) was investigated simultaneously. The activity of the *S. anginosus* BSU 1211 promoter was assessed in the *S. anginosus* type strain SK52 (Figure 4B). Promoter activity was surveyed after 2 h and was significantly higher when *egfp* was under the control of the SilX promoter than in the absence of putative promoter sequences. However, compared to the strong *cfb* promoter only low-grade activity could be observed. A supplementation with the signal peptide SilCR did not lead to a significantly increased promoter activity. Unfortunately, it was not possible to transform *S. anginosus* BSU 1211 with these constructs.

## Discussion

Bacterial CAAX proteases are intramembrane metalloproteases with various functions that are commonly present in bacteriocin gene clusters (Lux et al., 2007; Denapaite et al., 2010). In the context of bacteriocin production a connection of CAAX proteases to bacteriocin immunity has frequently been reported (Kjos et al., 2010). In this report we investigated the function of SilX, a CAAX protease of *S. anginosus*, which is involved in bacteriocin activity and the regulation of antimicrobial activity through the QS system *sil*.

Analysis of the protein sequences demonstrated classical CAAX protease motifs (Figure 1). To determine if SilX displays the predicted protease activity, the effect of HIV protease inhibitors on bacteriocin production was investigated. HIV protease inhibitors, like Nelfinavir, have previously been shown to inhibit bacterial CAAX proteases e.g., SagE of *S. pyogenes* (Maxson et al., 2015), which is involved in processing the hemolytic SagA peptide of the streptolysin S gene cluster. We could show that by incubation with Nelfinavir a dose dependent inhibition of bacteriocin production occurred. Also, Saquinavir had a dose-dependent effect on bacteriocin inhibition, however the effect was not as pronounced as for Nelfinavir. This might be explained by the difference in molecular weight. Saquinavir has a molecular weight of 766.95 Dalton and Nelfinavir of 567.78 Dalton. The smaller Nelfinavir might more easily diffuse across the cytoplasmic membrane and therefore lower concentrations are able to sufficiently inhibit SilX. Furthermore, Nelfinavir is more lipophilic than Saquinavir, which might also lead to a better uptake of the drug (Maxson et al., 2015). Nelfinavir is associated with higher intracellular concentrations than Saquinavir (Hoggard, 2003).

The dose-dependency of the effect clearly shows that the reduction in antimicrobial activity is due to SilX. In line with this, a SilX deletion mutant lost antimicrobial activity against the target strains of Angicin, suggesting that SilX is essential for bacteriocin production. It has previously been noted that CAAX proteases play an important role in bacteriocin production and CAAX knock out mutants in *Streptococcus pneumoniae* no longer display antimicrobial activity (Lux et al., 2007). Interestingly, bacteriocin production and thereby inhibition of target strains could be reestablished by addition of the signaling peptide SilCR, which is encoded in the adjacent QS system. These results are consistent with the hypothesis that SilX is responsible for processing and cleavage of SilCR into its mature configuration. SilCR has previously been demonstrated to induce bacteriocin production of *S. anginosus* (Vogel et al., 2021). The catalytic activity of CAAX proteases depends on two distinct sequence motifs EExxxR and FxxxH (Pei et al., 2011). The FxxxH motif is conserved in *S. anginosus* BSU 1211, while in the diglutamate motif the arginine residue is replaced by glutamine. However, both of these amino acid residues are polar, thus representing a conservative switch, that may preserve the catalytic function. Leading to the conclusion that SilX could still be able to process SilCR. If SilX processes SilCR, the question arises what the function of SilD and SilE might be. It would be possible that this export system is responsible for the processing and export of Angicin. Further experiments should aim at elucidating this question.

A similar mechanism of peptide maturation is proposed for SagE of *S. pyogenes* (Maxson et al., 2015). SagE is part of the Streptolysin S gene cluster mediating ß-hemolysis of this species. The ß-hemolysin itself is produced as a peptide protoxin (SagA), which is subsequently modified to become hemolytic. The authors could demonstrate that after an inhibition with HIV protease inhibitors, SagE is no longer able to process the pro-toxin of Streptolysin S (Maxson et al., 2015).

For the CAAX protease MorQ of *S. aureus* it is suggested that this enzyme facilitates the optimal processing or the export of the AIP of the *arg* system (Cosgriff et al., 2019). However, the precise mechanism is not yet understood. Another possible mechanism of action of SilX is an interaction with the histidine kinase SilB. Other CAAX proteases have been shown to interact with histidine kinases either increasing or decreasing their activity (Firon et al., 2013; Cosgriff et al., 2019). In *Staphylococcus aureus* in particular the interaction of a CAAX protease with the histidine kinase of a QS system has been demonstrated. It’s possible that SilX promotes SilB activity leading to the expression of bacteriocins. Consequently, SilB would be inactive in the SilX knock out mutant. However, this effect can be reversed by high SilCR concentrations, which are achieved by exogenously adding synthetic SilCR.

In other bacterial species several studies on CAAX proteases revealed functions connected to bacteriocin self immunity (Lux et al., 2007; Kjos et al., 2010). Immunity proteins protect producer strains against the detrimental effects of their own bacteriocins (Fimland et al., 2002). We found however no evidence that SilX is important for bacteriocin self-immunity in *S. anginosus*. The CAAX knock-out mutant strain we generated was not sensitive toward the Angicin-producing *S. anginosus* strain BSU 1211. Moreover *blp3.3* another gene of the *blp3* gene cluster of *S. anginosus* has already been shown to provide immunity functions (Vogel et al., 2021). In a next step promoter functionality and activity was surveyed. The *silX* gene expression is regulated by the *sil* locus and dependent on a transcription activator SilA (Belotserkovsky et al., 2009; Vogel et al., 2021). SilA belongs to the LytR regulator family that bind DNA as dimers at direct repeats. For *S. anginosus* we predicted a binding site composed of two 10 bp repeats and a 11 bp spacer. The same basic composition is also found for *S. pyogenes* and *S. intermedius* (Belotserkovsky et al., 2009; Mendonca et al., 2016). The SilA binding site was cloned in front of the reporter gene *egfp* to assess its functionality. Due to unknown reasons, it was not possible to transform *S. anginous* BSU 1211 with this construct. Alternatively, it was transformed into the *S. anginosus* type strain (SK 52), where an increased fluorescence could be observed in the strains harboring the putative SilX promoter sequence (Figure 4). However, compared to *cfb* promoter, which has been shown to induce high level gene expression in various streptococci (Aymanns et al., 2011), the SilX promoter shows only a moderate activity in this background and a supplementation with the signal peptide SilCR did not induce promoter activity. This may be explained by the fact that in *S. anginosus* SK52 the *silA* and *silB* gene are not present and SilCR needs this two-component system to efficiently alter gene expression (Belotserkovsky et al., 2009). Alternatively, this data could also indicate that SilX has a constitutive low-level expression that is independent from the *sil* system. If SilX processes SilCR, this would ensure the maturation of SilCR as soon as SilCR production is turned on. However, since the *sil* locus is not intact in *S. anginosus* SK52, it is difficult to draw definite conclusions, without investigating this construct in a *S. anginosus* strain harboring a *sil* locus.

CAAX proteases are often part of bacteriocin operons in streptococci. For example two CAAX proteases are found adjacent to the biosynthetic operon of gallocin from *Streptococcus gallolyticus* (Harrington et al., 2021; Proutière et al., 2021). The function of these proteins has so far not been investigated. In *S. mutans* the CAAX protease LsrS is described as protein with receptor-like function (Biswas and Biswas, 2014). For *S. pneumoniae* it was shown that the knockout of a CAAX protease led to a loss of antimicrobial achtivity (Lux et al., 2007). To our knowledge this is the first report to indicate that a streptococcal CAAX protease may be involved in processing the signal peptide of a quorum sensing system. It thus links a bacteriocin associated CAAX protease to the regulation of bacteriocin production rather than to bacteriocin self-immunity.

## Data Availability Statement

The raw data supporting the conclusions of this article will be made available by the authors, without undue reservation.

## Author Contributions

VV and BS designed this study. MF, MJ, AB, SM, and VV performed the experiments under the supervision of VV and BS. JM supported the experiments conducted with HIV inhibitors. VV wrote, and BS reviewed this manuscript. All authors contributed to the article and approved the submitted version.

## Conflict of Interest

The authors declare that the research was conducted in the absence of any commercial or financial relationships that could be construed as a potential conflict of interest.

## Publisher’s Note

All claims expressed in this article are solely those of the authors and do not necessarily represent those of their affiliated organizations, or those of the publisher, the editors and the reviewers. Any product that may be evaluated in this article, or claim that may be made by its manufacturer, is not guaranteed or endorsed by the publisher.

## Footnotes

1
http://www.ncbi.nlm.nih.gov/

2
https://blast.ncbi.nlm.nih.gov/Blast.cgi

3
https://www.snapgene.com/

4
http://www.clcbio.com
